# Antagonistic actions of boron against inhibitory effects of aluminum toxicity on growth, CO_2 _assimilation, ribulose-1,5-bisphosphate carboxylase/oxygenase, and photosynthetic electron transport probed by the JIP-test, of *Citrus grandis *seedlings

**DOI:** 10.1186/1471-2229-9-102

**Published:** 2009-08-01

**Authors:** Huan-Xin Jiang, Ning Tang, Jin-Gui Zheng, Li-Song Chen

**Affiliations:** 1 Institute of Horticultural Plant Physiology, Biochemistry and Molecular Biology, Fujian Agriculture and Forestry University, Fuzhou 350002, PR China; 2 College of Life Science, Fujian Agriculture and Forestry University, Fuzhou 350002, PR China; 3 College of Horticulture, Fujian Agriculture and Forestry University, Fuzhou 350002, PR China; 4 Biotechnology Center, Fujian Agriculture and Forestry University, Fuzhou 350002, PR China; 5 Fujian Key Laboratory for Plant Molecular and Cell Biology, Fujian Agriculture and Forestry University, Fuzhou 350002, PR China

## Abstract

**Background:**

Little information is available on the amelioration of boron (B) on aluminum (Al)-induced photosynthesis inhibition. Sour pummelo (*Citrus grandis*) seedlings were irrigated for 18 weeks with nutrient solution containing 4 B levels (2.5, 10, 25 and 50 μM H_3_BO_3_) × 2 Al levels (0 and 1.2 mM AlCl_3_·6H_2_O). The objectives of this study were to determine how B alleviates Al-induced growth inhibition and to test the hypothesis that Al-induced photosynthesis inhibition can be alleviated by B *via *preventing Al from getting into shoots.

**Results:**

B had little effect on plant growth, root, stem and leaf Al, leaf chlorophyll (Chl), CO_2 _assimilation, ribulose-1,5-bisphosphate carboxylase/oxygenase (Rubisco), Chl a fluorescence (OJIP) transient and related parameters without Al stress except that root, stem and leaf B increased with increasing B supply and that 50 μM B decreased slightly root dry weight. Al-treated roots, stems and leaves displayed a higher or similar B. B did not affect root Al under Al stress, but decreased stem and leaf Al level. Shoot growth is more sensitive to Al stress than root growth, CO_2 _assimilation, Chl, Rubisco, OJIP transient and most related parameters. Al-treated leaves showed decreased CO_2 _assimilation, but increased or similar intercellular CO_2 _concentration. Both initial and total Rubisco activity in Al-treated leaves decreased to a lesser extent than CO_2 _assimilation. Al decreased maximum quantum yield of primary photochemistry and total performance index, but increased minimum fluorescence, K-band, relative variable fluorescence at J- and I-steps. B could alleviate Al-induced increase or decrease for all these parameters. Generally speaking, the order of B effectiveness was 25 μM > 10 μM ≥ 50 μM (excess B) > 2.5 μM.

**Conclusion:**

We propose that Al-induced photosynthesis inhibition was mainly caused by impaired photosynthetic electron transport chain, which may be associated with growth inhibition. B-induced amelioration of root inhibition was probably caused by B-induced changes in Al speciation and/or sub-cellular compartmentation. However, B-induced amelioration of shoot and photosynthesis inhibition and photoinhibitory damage occurring at both donor and acceptor sides of photosystem II could be due to less Al accumulation in shoots.

## Background

Acid soils comprise up to 50% of the world's potentially arable lands. In many acid soils through the tropics and subtropics, Al toxicity is a major factor limiting crop productivity [1]. Al^3+ ^is the most important rhizotoxic Al species and is abundant at pH 4.0 – 4.5 [2-4]. Once Al is inside the plants, it is likely to be present as Al(OH)_3_, which is structurally similar to B(OH)_3 _[5]. Evidence shows that root apex, and more specifically the distal part of the transition zone within the apex, is the primary site for Al toxicity [6-9]. The primary symptom of Al toxicity is a rapid inhibition of root growth, which occurs within minutes upon exposure to Al stress [1]. The rapidity of root growth inhibition means that Al first inhibits root cell expansion and elongation, prior to inhibiting cell division [5,10]. Al is assumed to exert its toxic effect in the apoplast through interaction with the negative binding sites of the cell walls, primarily pectin of root epidermal and cortical cells [11,12]. B deficiency is a widespread problem in many agricultural crops, including *Citrus *spp. B deficiency occurs most frequently on course-textured soils with low organic matter status. It is also a problem in acid soils in humid climates where B content is low because of high leaching losses [13]. Like Al, B also primarily inhibits root growth through limiting cell elongation rather than cell division, which is probably the secondary response of the root meristematic region to B deficiency [14]. Evidence shows that the predominant function of B is in the formation of primary cell walls, where it cross-links the pectic polypectic polysaccharide rhamnogalacturonan II (RG-II) [15,16]. Lukaszewski and Blevins [17] reported that root growth inhibition in B-deficient or Al-toxic squash plants (*Cucurbita pepo*) could be a consequence of a disrupted ascorbate metabolism. Based on the similarities of the molecules and of the symptom characteristic for Al-toxic and B-deficient plants, it has been proposed that Al may exert its toxic effect by inducing B deficiency [4]. LeNoble et al. [18,19] showed that supraoptimal B concentration prevented Al-induced inhibition of root growth of squash in solution culture and of alfalfa (*Medicago sativa*) in soil culture. B also alleviate Al toxicity in apple rootstock P22 [20], common bean (*Phaseolus vulgaris*) [21], pea (*Pisum sativum*) [22]. However, other investigations in wheat (*Triticum aestivum*) [23] and maize (*Zea mays*) [24] did not find evidence that B was capable of ameliorating Al toxicity. Recently, Corrales et al. [25] reported that B alleviated Al toxicity in both cucumber (*Cucumis sativus*) and maize, but only in the former was B able to protect against Al-induced inhibition of root elongation. Evidence suggests that B decreases the binding sites for Al in the cell walls, and hence Al toxicity [21,22]. In the other hand, the cross-linking RG-II by B ester results in a stable of network of cell walls with decreased pore sizes [16,26], thus hampering Al from getting into contact with sensitive targets at the plasma membrane and/or symplasm [25].

LeNoble et al. [18] found that protection against Al inhibition with B was also apparent for shoot growth of Al-stressed squash in solution culture. Recently, Yu et al. [22] reported that B alleviated the chlorosis-symptoms of Al toxicity, and prevented the decrease in Chl concentration and the inhibition of shoot growth after prolonged exposure to Al stress, which was accompanied by a lower Al level in shoots. Therefore, B may alleviate Al-induced inhibition of photosynthesis after prolonged exposure to Al stress. To our knowledge, very little information is available on the ameliorative effects of B on Al-induced inhibition of photosynthesis.

Citrus belongs to evergreen subtropical fruit trees and is cultivated in humid and subhumid of tropical, subtropical, and temperate regions of the world mainly on acid soils. High Al and low B are frequently observed in citrus plantations. Although the effects of Al toxicity or B deficiency on citrus growth and CO_2 _assimilation have been studied by a few researchers [27-32], Al toxicity and low B are almost always investigated separately as independent factors. In this paper, we investigated the effects of Al and B interactions on plant growth, the concentrations of Al and B in roots, stems and leaves, and leaf CO_2 _assimilation, Rubisco (EC 4.1.1.39) and photosynthetic electron transport probed by the JIP-test, of sour pummelo (*Citrus grandis*), an Al-sensitive rootstock used in pummelo cultivation. The objectives of this study were to determine how B alleviates Al-induced inhibition of root and shoot growth and to test the hypothesis that Al-induced inhibition of photosynthesis can be alleviated by B *via *preventing Al from getting into shoots.

## Results

### Seedling growth

**Figure 1:**
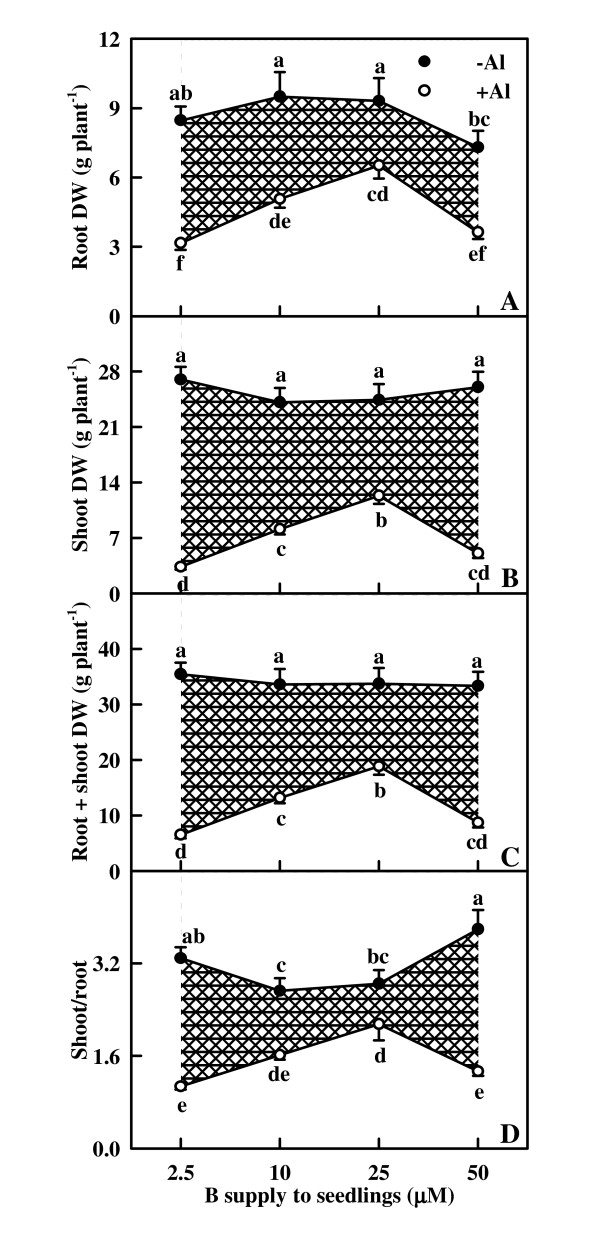
**Effects of Al and B interactions on root (A), shoot (B) and root + shoot (C) DW, and shoot/root ratio (D) of *Citrus grandis *seedlings**. Diagonal cross area quantifies the Al effect under different B supply. Each point is mean of 8 – 15 replicates with standard error. Difference among eight treatments was analyzed by 2 (Al levels) × 4 (B levels) ANOVA. (A) *P *values for Al, B, and the interaction between the two were < 0.0001, < 0.0001 and 0.3905; (B), (C), and (D) *P *values for Al, B, and the interaction between the two were all < 0.0001. Different letters indicate significant differences among eight treatments at *P *< 0.05.

B did not affect significantly root (Fig. 1A), shoot (Fig. 1B) and root + shoot (Fig. 1C) DW over the range of B supply in the absence of Al except that 50 μM B supply decreased slightly the root DW (Fig. 1A), whereas they increased as B supply increased from 2.5 to 25 μM, then decreased at the highest B supply under Al stress (Fig. 1A–C). Root, shoot and root + shoot DW were lower in +Al seedlings than in -Al ones at each given B level (Fig. 1A–C).

Shoot/root ratio was higher in 2.5 and 50 μM B-treated seedlings than in 10 and 25 μM B-treated ones in the absence of Al, whereas increased as B supply increased from 2.5 to 25 μM, then decreased at the highest B supply under Al stress. Shoot/root ratio was higher in -Al seedlings than in +Al ones at each given B level (Fig. 1D).

### Al and B concentrations in roots, stems and leaves

**Figure 2:**
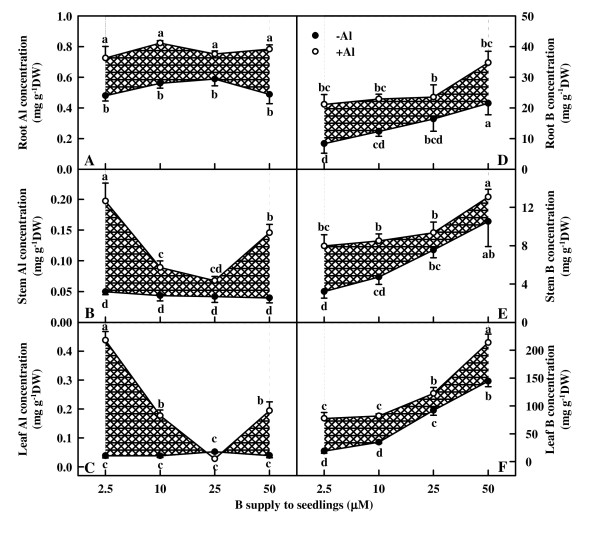
**Effects of Al and B interactions on the concentrations of Al and B in *Citrus grandis *roots, stems and leaves**. Diagonal cross area quantifies the Al effect under different B supply. Each point is mean of 4 – 5 replicates with standard error. Difference among eight treatments was analyzed by 2 (Al levels) × 4 (B levels) ANOVA. *P *values for Al, B, and the interaction between the two were 0.0001, 0.3206 and 0.3148 (A); < 0.0000, < 0.0000 and 0.0001 (B); < 0.0001, < 0.0001 and < 0.0001 (C); 0.0002, 0.0066 and 0.8568 (D); and 0.0006, 0.0001 and 0.7807 (E); 0.0001, 0.0001 and 0.2002 (F); respectively. Different letters indicate significant differences among eight treatments at *P *< 0.05.

Al increased root Al concentration, whereas B did not affect significantly root Al concentration (Fig. 2A). Al concentration of -Al stems and leaves did not change significantly in response to B, whereas that of +Al stems and leaves decreased with increasing B supply from 2.5 to 25 μM, then increased at the highest B supply. Al concentration was significantly higher in +Al stems and leaves than in -Al ones except for a similar Al concentration between the two under 25 μM B (Fig. 2B and 2C).

Root, stem and leaf B concentration increased with increasing B supply whether seedlings were treated with or without Al. Al-treated roots, stems and leaves displayed a higher or similar B concentration (Fig. 2D–F).

### Leaf Chl, root and leaf total soluble protein

**Figure 3:**
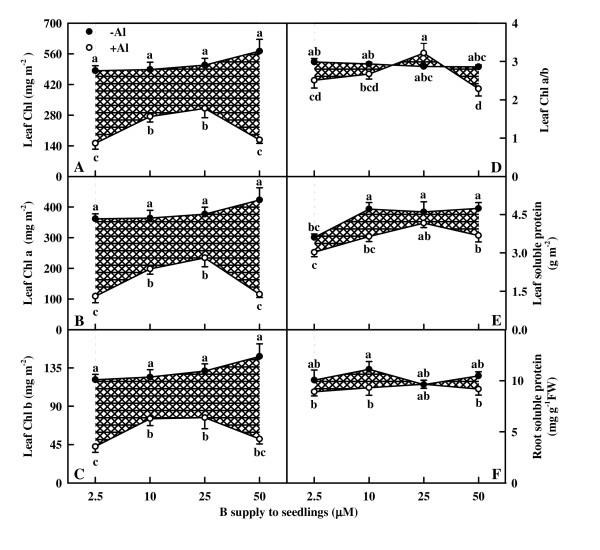
**Effects of Al and B interactions on Chl (A), Chl a (B) and Chl b (C) concentrations and Chl a/b ratio (D) of leaves, and total soluble protein concentration of roots (E) and leaves (F) in *Citrus grandis *seedlings**. Diagonal cross area quantifies the Al effect under different B supply. Each point is mean of 4 – 5 replicates with standard error. Difference among eight treatments was analyzed by 2 (Al levels) × 4 (B levels) ANOVA. *P *values for Al, B, and the interaction between the two were < 0.0001, 0.0016 and 0.0557 (A); < 0.0001, 0.0076 and 0.0311 (B); < 0.0001, 0.0339 and 0.2040 (C); 0.0314, 0.0255 and 0.0164 (D); < 0.0001, 0.0001 and 0.2386 (E); 0.0236, 0.6780 and 0.5181 (F), respectively. Different letters indicate significant differences among eight treatments at *P *< 0.05.

Al decreased leaf Chl, Chl a and Chl b concentrations at each given B level. The concentrations of Chl, Chl a and Chl b did not change significantly in response to B in the absence of Al, while increased with increasing B supply from 2.5 to 10 μM under Al stress, then remained unchanged with further increasing B supply or decreased at the highest B supply (Fig. 3A–C). Chl a/b ratio remained unchanged over the range of P supply examined in the absence of Al, whereas increased as B supply increased from 2.5 to 25 μM under Al stress, then decreased at the highest B supply. Chl a/b ratio was lower in +Al leaves than in -Al ones under 2.5 or 50 μM B, but there was no significant difference between the two under 10 or 25 μM B (Fig. 3D).

Foliar total soluble protein concentration increased as B supply increased from 2.5 to 10 μM in the absence of Al and from 2.5 to 25 μM under Al stress, then remained unchanged with further increasing B supply. Total soluble protein concentration was slightly lower in +Al leaves than in -Al ones under 10 or 50 μM B, but there was no significant difference between the two under 2.5 or 25 μM B (Fig. 3E). B did not affect significantly root total soluble protein concentration whether seedlings were treated with or without Al and there was no significant difference between roots treated with or without Al over the range of B supply except that the protein concentration was slightly lower in +Al roots than in -Al ones under 10 μM B (Fig. 3F).

### Leaf gas exchange and Rubisco

**Figure 4:**
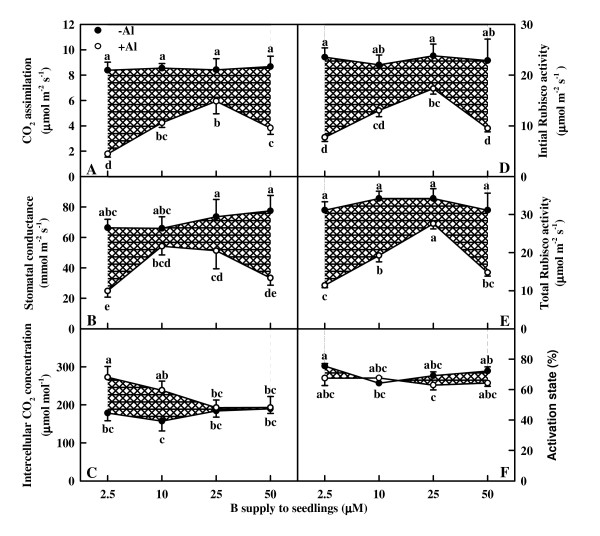
**Effects of Al and B interactions on CO_2 _assimilation (A), stomatal conducance (B), intercellular CO_2 _concentration (C), initial Rubisco activity (D), total Rubisco activity (E), and activation state (F) in *Citrus grandis *leaves**. Diagonal cross area quantifies the Al effect under different B supply. Each point is mean of 4 – 5 replicates with standard error. Difference among eight treatments was analyzed by 2 (Al levels) × 4 (B levels) ANOVA. *P *values for Al, B, and the interaction between the two were < 0.0001, 0.0121 and 0.0492 (A); 0.0308, 0.5072 and 0.1252 (B); 0.0041, 0.3548 and 0.1155 (C); < 0.0001, 0.1192 and 0.2326 (D); < 0.0001, 0.0016 and 0.1244 (E); 0.0575, 0.2836 and 0.2166 (F), respectively. Different letters indicate significant differences among eight treatments at *P *< 0.05.

B did not affect significantly CO_2 _assimilation, stomatal conductance and intercellular CO_2 _concentration without Al stress (Fig. 4A–C). CO_2 _assimilation in +Al leaves increased as B supply increased from 2.5 to 25 μM, then decreased at the highest B supply (Fig. 4A). Stomatal conductance in +Al leaves increased as B supply increased from 2.5 to 10 μM, then did not change significantly with further increasing B supply (Fig. 4B). Intercellular CO_2 _concentration in +Al leaves decreased as B supply increased from 2.5 to 25 μM, then remained unchanged at the highest B supply (Fig. 4C). Al-treated leaves displayed a lower CO_2 _assimilation (Fig. 4A), a lower or similar stomatal conductance (Fig. 4B), but a higher or similar intercellular CO_2 _concentration (Fig. 4C).

Both initial and total Rubisco activity did not change significantly in response to B in the absence of Al, but increased as B supply increased from 2.5 to 25 μM under Al stress, then decreased at the highest B supply. Both initial and total Rubisco activity was higher in -Al leaves than in +Al ones except that there was no significant difference between the two under 25 μM B (Fig. 4D and 4E). No significant difference was found in Rubisco activation state among Al and B combinations except for a slight decrease in the combinations of 10 μM B + 0 mM Al and 25 μM B + 1.2 mM Al (Fig. 4F).

### Leaf OJIP transients and related parameters

**Figure 5:**
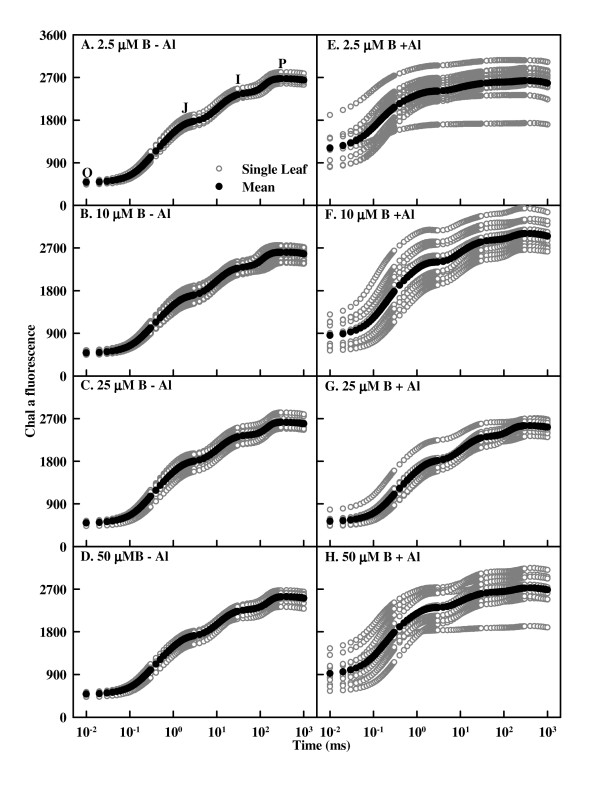
**Effects of Al and B interactions on high irradiance actinic-light-induced OJIP transients of dark-adapted *Citrus grandis *leaves plotted on a logarithmic time scale (0.01 to 1 s)**. Gray circles are single measurement and black circles are mean transients of all measured samples. Al increased the heterogeneity of samples, which was the lowest under 25 μM B and the highest under 2.5 μM B.

OJIP transients from -Al leaves showed little change in response to B (Fig. 5A–D). Al increased the heterogeneity of samples, which decreased as B supply increased from 2.5 to 25 μM, then increased at the highest B supply (Fig. 5E–H). Al-treated leaves showed increased O-step and similar P-step under 2.5 μM or 50 μM B (Fig. 5E and 5F), whereas both the O- and the P-steps increased under 10 μM B (Fig. 5H).

**Figure 6:**
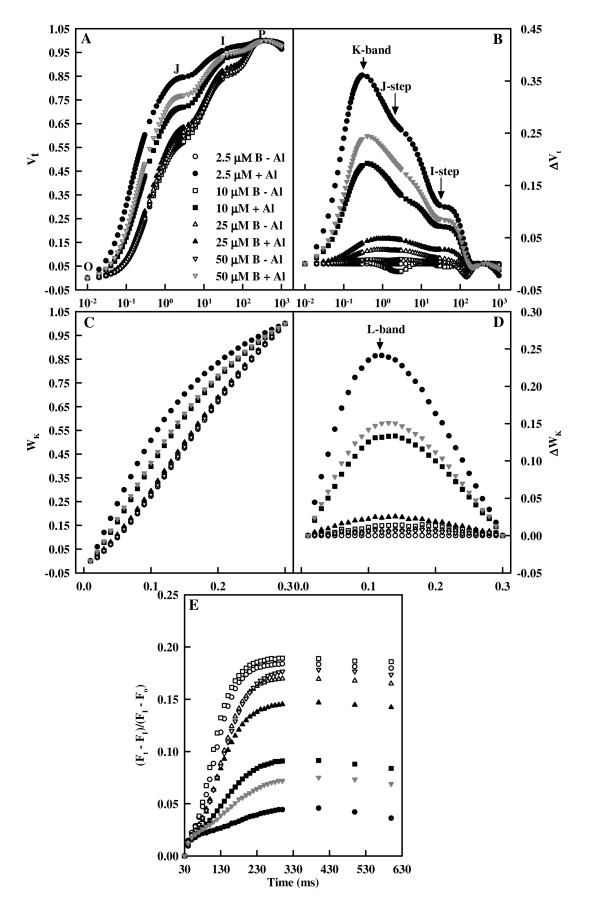
**Effects of Al and B on mean OJIP transients expressed as the kinetics of relative variable fluorescence**: (A) between F_o _and F_m_: V_t _= (F_t _- F_o_)/(F_m _- F_o_) and (B) the differences of the eight samples to the reference sample treated with 2.5 μM B + 0 mM Al (ΔV_t_), (C) between F_o _and F_300 μs_: W_K _= (F_t _- F_o_)/(F_300 μs _- F_o_) and (D) the differences of the eight samples to the reference sample (ΔW_K_), (D) IP phase: (F_t _- F_o_)/(F_I _- F_o_) - 1 = (F_t _- F_I_)/(F_I _- F_o_) in dark-adapted *Citrus grandis *leaves.

Fig. 6A and 6B shows the kinetics of relative variable fluorescence at any time V_t _= (F_t _- F_o_)/(F_m _- F_o_) and the differences of all normalized transients minus 2.5 μM B + 0 mM Al treated transient (ΔV_t_). The differences revealed one positive K-band (300 μs) and two positive steps: the J- and I-steps. The positive K-band, I- and J-steps were the most pronounced in 2.5 μM B-treated leaves, followed in 50, 10 and 25 μM B-treated leaves under Al stress, whereas B had little effect on them without Al stress. Fig. 6C and 6D depicts the relative variable fluorescence between F_o _and F_300 μs _(W_K_) and the differences of eight mean transients minus 2.5 μM B + 0 mM Al treated mean transient (ΔW_K_). Al resulted in an increase in the L-band, whose amplitude decreased as B supply increased from 2.5 to 25 μM under Al stress, then increased at the highest B supply. Al decreased the maximum amplitude of IP phase, which showed little change in response to B without Al stress, whereas increased as B supply increased from 2.5 to 25 μM under Al stress, then decreased at the highest B supply (Fig. 6E).

**Figure 7:**
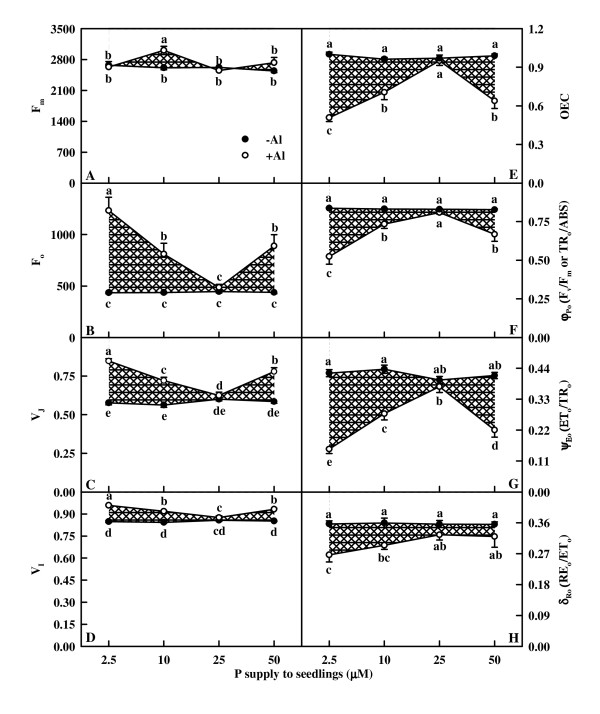
**Effects of Al and B interactions on F_m _(A), F_o _(B), V_J _(C), V_I _(D), OEC (E), φ_Po_(F), ψ_Eo _(G) and δ_Ro _(H) of dark-adapted *Citrus grandis *leaves**. Each point is mean of 8 – 10 replicates with standard error. Diagonal cross area quantifies the Al effect under different B supply. Difference among eight treatments was analyzed by 2 (Al levels) × 4 (B levels) ANOVA. (A) *P *values for Al, B, and the interaction between the two were 0.0135, 0.0248 and 0.0085, respectively; (B) – (G) *P *values for Al, B, and the interaction between the two were all < 0.0001; (H) *P *values for Al, B, and the interaction between the two were < 0.0001, 0.1995 and 0.2667, respectively. Different letters indicate significant differences among eight treatments at *P *< 0.05.

**Figure 8:**
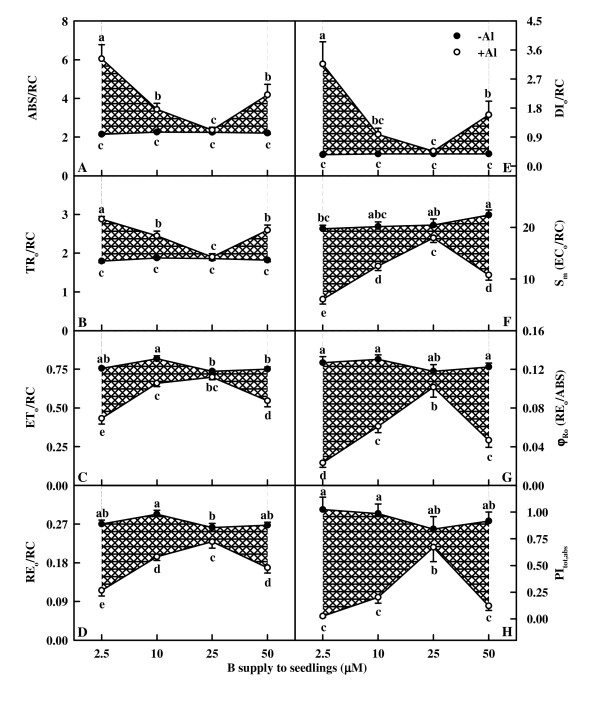
**Effects of Al and B interactions on ABS/RC (A), TR_o_/RC (B), ET_o_/RC (C), RE_o_/RC (D), DI_o_/RC (E), S_m _(EC_o_/RC, F), φ_Ro_(G) and PI_tot,abs _(H) of dark-adapted *Citrus grandis *leaves**. Diagonal cross area quantifies the Al effect under different B supply. Each point is mean of 8 – 10 replicates with standard error. Difference among eight treatments was analyzed by 2 (Al levels) × 4 (B levels) ANOVA. (A) – (C) and (F) – (G) *P *values for Al, P, and the interaction between the two were all < 0.0001; (D) – (E) *P *values for Al, P, and the interaction between the two were < 0.0000, < 0.0000 and 0.0001; (H) *P *values for Al, P, and the interaction between the two were 0.0216, 0.0000 and 0.0002, respectively. Different letters indicate significant differences among eight treatments at *P *< 0.05.

As shown in Fig. 7 and 8, all fluorescence parameters did not change significantly in response to B without Al-stress except for a slight decrease for EC_o_/RC in 2.5 μM B-treated leaves (Fig. 8F) and a slight increase for ET_o_/RC (Fig. 8C) and RE_o_/RC (Fig. 8D) in 10 μM B-treated leaves. No significant difference was found in all these parameters between +Al and -Al leaves under 25 μM B except that RE_o_/RC (Fig. 8D) and EC_o_/RC (Fig. 8F) were lower in +Al leaves than in -A ones. No significant difference was found in F_m _(Fig. 7A) among Al and B combinations except for an increase in leaves treated with 10 μM B + 1.2 mM Al. Under 2.5, 10 or 50 μM B, Al-treated leaves had increased F_o _(Fig. 7B), V_J _(Fig. 7C), V_I _(Fig. 7D), ABS/RC (Fig. 8A), TR_o_/RC (Fig. 8B), DI_o_/RC (Fig. 8E) and deactivation of OEC (Fig. 7E), but decreased TR_o_/ABS (Fig. 7F), ET_o_/TR_o _(Fig. 7G), RE_o_/ET_o _(Fig. 7H), ET_o_/RC (Fig. 8C), RE_o_/RC (Fig. 8D), EC_o_/RC (Fig. 8F), RE_o_/ABS (Fig. 8G) and PI_tot,abs _(Fig. 8H). The extent of increase or decrease for the 15 parameters was higher in 2.5 μM B-treated leaves than in 10 or 50 μM B-treated ones, but similar between 10 and 50 μM B-treated leaves except that the extent of decrease in ET_o_/TR_o _(Fig. 7G) and ET_o_/RC (Fig. 8C) or increase in V_J_(Fig. 7D) was less in 10 μM B-treated leaves than in 50 μM B-treated ones under Al-stress.

### Leaf initial Rubisco activity, maximum amplitude of IP phase and PI_tot,abs _in relation to CO_2 _assimilation and shoot DW

**Figure 9:**
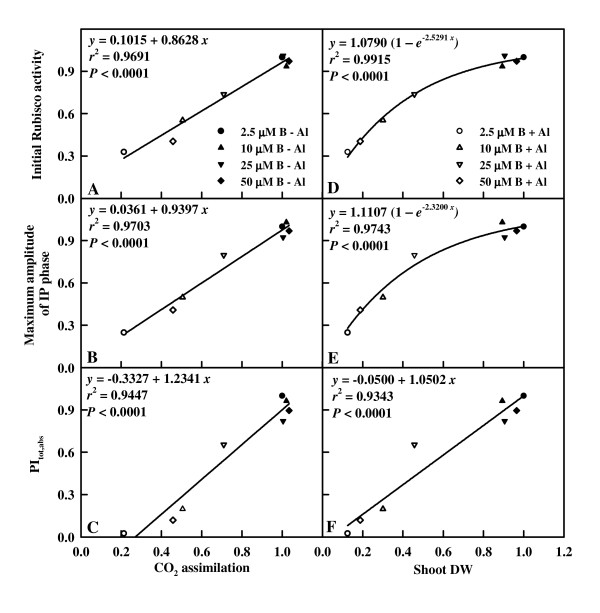
**Initial Rubisco activity (A, D), maximum amplitude of IP phase (B, E) and PI_tot,abs _(C, F) in relation to leaves CO_2 _assimilation and shoot DW in *Citrus grandis *seedlings**. All the values were expressed relative to the sample treated with 2.5 μM B + 0 mM Al set as 1. Maximum amplitude of IP phase = (F_m _- F_o_)/(F_I _- F_o_) - 1.

Leaf CO_2 _increased with increasing leaf initial Rubisco activity (Fig. 9A), maximum amplitude of IP phase (Fig. 9B) and PI_tot,abs _(Fig. 9C), respectively. Leaf initial Rubisco activity (Fig. 9D), maximum amplitude of IP phase (Fig. 9E) and PI_tot,abs _(Fig. 9F) increased with increasing shoot DW, respectively.

## Discussion

The present work, like that of previous workers [18,19,22] indicates that B prevent the inhibition of root and shoot growth (Fig. 1A–C) and the decrease in Chl, Chl a and Chl b concentrations (Fig. 3A–C) under Al stress. The ameliorative effects of B was not brought about by an increase in the B concentration of roots, stems and leaves, because B concentration was not lower in +Al roots, stems and leaves than in -Al ones (Fig. 2D–F). This agrees with early reports that Al did not affect B concentration in the roots of soybean (*Glycine max*) [33] and maize [24]. Our results showed that the sequence of the ameliorative effect of B on growth inhibition and Chl decrease in +Al seedlings was 25 μM B > 10 μM B ≥ 50 μM B > 2.5 μM B (Fig. 1A–C and 3A–C), indicating that Al-induced growth inhibition is not due to Al-induced B deficiency. Corrales et al. [25] showed that Al increased the concentration of reduced glutathione in roots of maize plants growing with adequate B supply but not in those growing in excess B, which, in turn, caused extensive cell damage in the root tips of maize plants even in the absence of Al. The lower root DW in 50 μM B + 0 mM Al treated seedlings (Fig. 1A) implies that these plants received excess B. This would explain why the ameliorative effect of 50 μM B was lower than that of 25 μM B, because +Al roots, stems and leaves displayed higher or similar B concentration (Fig. 2D–F). No difference for root Al concentration among B treatments (Fig. 2A–C) indicates that the B-induced amelioration of root inhibition was probably caused by B-induced changes in Al speciation and/or sub-cellular compartmentation [25] rather than by less Al accumulation in root tips [22,33]. Our finding that Al concentration was the highest in 2.5 μM B-treated stems and leaves under Al stress, followed by 10, 50 and 25 μM B-treated ones (Fig. 2A–C) indicates that B-induced amelioration of shoot inhibition could be due to less Al accumulation in shoots.

The higher or similar intercellular CO_2 _concentration in +Al leaves indicates that Al-induced decrease in CO_2 _assimilation (Fig. 4A and 4C) is primarily caused by non-stomatal factors, as earlier reported for citrus [27,28,30] and sorghum (*Sorghum bicolor*) [34]. The finding that Al decreased initial and total Rubisco activity except for no difference for total Rubisco activity between Al treatments under 25 μM B (Fig. 4D and 4E) contrasts with previous reports that Al-induced decrease in CO_2 _assimilation in sour pummelo [30] and in an Al-tolerant rootstock 'Cleopatra' tangerine (*Citrus reshni*) [28,35] was unaccompanied by decreased total Rubisco activity. In the other one study, we found that both the initial and total Rubisco activity was lower in 1.2 mM Al + 10 μM B treated leaves than in 0 mM Al + 10 μM B treated ones under 50 or 100 μM P, whereas there was no difference between the two under 250 or 500 μM P (data not shown). It is worth noting that in previous experiments, the nutrient solution for 'Cleopatra' tangerine [28] and sour pummelo [30] contained 250 μM P + 50 μM B and 100 μM P + 46 μM B, respectively. Thus, it appears that the influence of Al on Rubisco activity depends on P and B concentrations and citrus species. The finding that CO_2 _assimilation decreased with decreasing initial Rubisco activity (Fig. 9A) does not implies that the decrease in initial and total Rubisco activity in response to Al is the primary factor limiting CO_2 _assimilation, because Rubisco activity decreased to a lesser extent than CO_2 _assimilation (Fig. 4A, 4D, 4E and 9A). Our results showed that Chl concentration was lower in 50 μM B + 1.2 mM Al treated leaves than in 10 μM B + 1.2 mM Al treated ones (Fig. 3A), but there was no difference in CO_2 _assimilation between the two (Fig. 4A), suggesting that Al-induced decrease in CO_2 _assimilation cannot be attributed to a decrease in Chl concentration. This is also supported by our data that there was a greater excess of absorbed light energy in +Al leaves than in -Al ones, as indicated by increased DI_o_/RC (Fig. 8E), DI_o_/ABS, and DI_o_/CS_o _(data not shown).

The Al-induced L-band at ca. 110 – 140 μs (Fig. 6D) agrees with the results obtained for Al-stressed [30] and B-stressed [32] sour pummelo leaves. According to the Grouping Concept [36], the less pronounced L-band in +Al leaves with 10, 25 and 50 μM B compared with 2.5 μM B indicates that B supply enhances the grouping of PSII units and the energy exchange between the independent PSII units. Because the grouped conformation is more stable than the ungrouped one, the decreased grouping implies that the PSII units of +Al leaves have lost stability and become more fragile. This would explain why Al increased the heterogeneity of the samples (Fig. 5).

The decrease of F_v_/F_m _(TR_o_/ABS, Fig. 7F) in +Al leaves was mainly caused by an increase in F_o _(Fig. 5 and 7A–B), as previously found for B-excess sour pummelo leaves [32]. An increase in F_o _is thought to indicate photoinhibitory damage [37]. The higher TR_o_/ABS and the lower F_o _in +Al leaves with 10, 25 and 50 μM B compared with 2.5 μM B indicates that B can alleviate Al-induced photoinhibitory damage.

The striking Al-toxic effect was the big increase in K-band, especially in leaves with 2.5 μM B (Fig. 6B), which agrees with previous results found for Al-stressed [30] and B-stressed [32] sour pummelo leaves. This suggests that the OEC is damaged [38,39] and the energetic connectivity between photosynthetic units is changed [38]. This is also supported by the data showing that +Al leaves had increased deactivation of OEC (Fig. 7E) and less energy exchange between independent PSII units, as indicated by the positive L-band (Fig. 6D). The increased V_J _and V_I _(Fig. 7C and 7D) and the decreased maximum amplitude of IP phase (Fig. 6D) indicate that the acceptor side of PSII become more reduced under Al stress, but the acceptor side of PSI become more oxidized. Al-induced photoinhibitory damage at PSII acceptor is also supported by the fact that Al resulted in a decrease in F_v _(F_v _= F_m _- F_o_) and an increase in F_o _(Fig. 5 and 7B), which is the characteristic of photoinhibitory damage at PSII acceptor side [40]. The less pronounced K-band, J- and I-steps (Fig. 6A and 6B) and the less deactivation of OEC (Fig. 7E) in +Al leaves with 10, 25 and 50 μM B compared with 2.5 μM B indicate that B can alleviate Al-induced photoinhibitory damage occurring at both the donor (i.e., the OEC) and the acceptor sides of PSII.

Our results showed that Al decreased the total electron carriers per RC (EC_o_/RC; Fig. 8F), the yields (TR_o_/ABS, RE_o_/ET_o_, ET_o_/TR_o _and RE_o_/ABS; Fig. 7F, 7G, 7H and 8G), the fluxes (RE_o_/RC; Fig. 8D) and the fractional reduction of the PSI end electron acceptors, as indicated by the decreased maximum amplitude of IP phase (Fig. 6D), and damaged all of the photochemical and non-photochemical redox reactions, as indicated by the decreases in PI_tot,abs _(Fig. 8H). This suggests that Al impairs the whole photosynthetic electron transport chain up to the reduction of end acceptors of PSI, thus limiting the production of reducing equivalents. Our finding that the decrease in the eight parameters mentioned above under Al stress was less pronounced in leaves treated with 10, 25 and 50 μM B than with 2.5 μM indicates that B can alleviate the toxicity of Al on whole photosynthetic electron transport chain. Regressive analysis showed that CO_2 _assimilation decreased with decreasing maximum amplitude of IP phase (Fig. 9B) and PI_tot,abs _(Fig. 9C), respectively, and that IP phase (Fig. 9E) and PI_tot,abs _(Fig. 9F) decreased with decreasing shoot DW, respectively. Our results showed that shoot growth was more sensitive to Al toxicity than root growth, CO_2 _assimilation, OJIP transient and most related parameters (Fig. 1, 4A, 5, 7 and 8). Therefore, we conclude that the decreased photosynthetic electron transport capacity, which may be associated with growth inhibition, is probably the primary factor contributing to decreased CO_2 _assimilation in Al-treated leaves.

The increased energy dissipation, as indicated by increased DI_o_/RC (Fig. 8E) in +Al leaves agreed with the increased requirement for dissipating more excess excitation energy existed in +Al leaves due to less utilization of the absorbed light in photosynthetic electron transport, as indicated by the decrease in EC_o_/RC (Fig. 8F), ET_o_/RC (Fig. 8C), RE_o_/RC (Fig. 8D), RE_o_/ABS (Fig. 8G) and PI_tot,abs _(Fig. 8H). The less impaired photosynthetic electron transport chain in leaves treated with 10, 25 and 50 μM B than with 2.5 μM under Al stress would explained why DI_o_/RC (Fig. 8E) increased to a lesser extent in the former than in the latter.

## Conclusion

The present work demonstrates that shoot growth is more sensitive to Al toxicity than root growth, CO_2 _assimilation, Chl, Rubisco, OJIP transient and most related parameters. We propose that Al-induced decrease in CO_2 _assimilation was mainly caused by impaired photosynthetic electron transport chain, which may be associated with growth inhibition. No difference for Al concentration in +Al roots among B treatments indicates that B-induced amelioration of root inhibition was probably caused by B-induced changes in Al speciation and/or sub-cellular compartmentation rather than by less Al accumulation in roots. However, B-induced amelioration of shoot and photosynthesis inhibition, Chl comcentration and Rubisco activity decrease, and photoinhibitiory damage occurring at both the donor and acceptor sides of PSII could be due to less Al accumulation in shoots, because B decreased stem and leaf Al concentration under Al stress. The ameliorative effects of B was not brought about by an increase in the B concentration, because +Al roots, stems and leaves displayed a higher or similar B concentration. This would explain why the ameliorative effect of 25 μM B is better than that of 50 μM B (excess B).

## Methods

### Plant culture and treatments

This study was conducted outdoors from February to November 2007 at Fujian Agriculture and Forestry University (FAFU). Seeds of sour pummelo (*Citrus grandis *(L.) Osbeck) were germinated in sand in plastic trays. Five weeks after germination, uniform seedlings with single stem were selected and transported to 6 L pots containing sand. Seedlings, three to a pot, were grown in a greenhouse under natural photoperiod at FAFU. Each pot was supplied with 500 mL of nutrient solution every two days. The nutrient solution contained the following macronutrients (in mM): KNO_3_, 1; Ca(NO_3_)_2_, 1; KH_2_PO_4_, 0.1; and MgSO_4_, 0.5; and micronutrients (in μM): H_3_BO_3_, 10; MnCl_2_, 2; ZnSO_4_, 2; CuSO4, 0.5; (NH_4_)_6_Mo_7_O_24_, 0.065; and Fe-EDTA, 20. Six weeks after transplanting, the treatment was applied for 18 weeks: until the end of the experiment, each pot was supplied daily until dripping with nutrient solution containing four B levels (2.5, 10, 25 and 50 μM H_3_BO_3_) × two Al levels [0(-Al) and 1.2 mM AlCl_3_·6H_2_O (+Al)]. The pH of the nutrient solutions was adjusted to 4.1 – 4.2 using HCl or NaOH. At the end of the experiment, the fully expanded (about 7-weeks-old) leaves from different replicates and treatments were chosen for all the measurements. For the determination of Rubisco, Chl and protein, leaf discs (0.61 cm^2 ^in size) were collected at noon in full sun, frozen in liquid nitrogen, and stored at -80°C until assayed.

### Measurement of plant DW

At the end of the experiment, 8 – 15 plants per treatment from different pots were harvested. The plants were divided into roots and shoots. The plant material was dried at 80°C for 48 h and DW measured [28].

### Assays of Chl, total soluble protein, total B and total Al

Leaf Chl was extracted and measured according to Lichtenthaler [41]. Leaf and root total soluble protein was determined according to Bradford [42]. Root, stem and leaf total B was determined according to Kowalenko and Lavkulich [43]. Root, stem and leaf total Al was determined colorimetrically by the aluminon method [44].

### Leaf gas exchange measurements

Measurements were made by a CI-301PS portable photosynthesis system (CID, WA, USA) at ambient CO_2 _concentration with a photosynthetic photon flux of 1300 μmol m^-2 ^s^-1 ^between 9:30 and 11:00 on a clear day [28,45]. During measuring, leaf temperature and relative humidity were 28 ± 0.2°C and 76 ± 0.5%, respectively.

### Leaf Rubisco activity measurements

Rubisco was extracted according to Chen et al. [28]. Rubisco activity was assayed according to Cheng and Fuchigami [45] with some modifications. For initial activity, 50 μL of sample extract was added to a cuvette containing 900 μL of assay solution, immediately followed by adding 50 μL of 10 mM RuBP, then mixing well. The change of absorbance at 340 nm was monitored for 40 s. For total activity, 50 μL of 10 mM RuBP was added 15 min later after 50 μL of sample extract was combined with 900 μL of assay solution to fully activate all the Rubisco. The assay solution for both initial and total activity measurements, whose final volume was 1 mL, contained 100 mM Hepes-KOH (pH 8.0), 25 mM KHCO_3_, 20 mM MgCl_2_, 3.5 mM ATP, 5 mM phosphocretaine, 5 units NAD-glyceraldehyde-3-phosphate dehydrogenase (NAD-GAPDH, EC 1.2.1.12), 5 units 3-phosphoglyceric phospokinase (PCK, EC 2.7.2.3), 17.5 units creatine phosphokinase (EC 2.7.3.2), 0.25 mM NADH, 0.5 mM RuBP, and 50 μL sample extract. Rubisco activation state was calculated as the ratio of initial activity to total activity.

### Measurements of leaf OJIP transients

OJIP transient was measured by a Handy Plant Efficiency Analyser (Handy PEA, Hansatech Instruments Limited, Norfolk, UK) according to Strasser et al. [46]. The transient was induced by red light of about 3400 μmol m^-2 ^s^-1 ^provided by an array of 3 light-emitting diodes (peak 650 nm) that focused on the leaf surface to give homogenous illumination over the exposed area of the leaf (4 mm in diameter). Initially, data are sampled at 10 μs intervals for the first 300 μs. The time resolution of digitization is then switched to slower acquisition rates as the kinetics of the fluorescence signal slow. All the measurements were done with 3 h dark-adapted plants at room temperature.

### JIP test

OJIP was analyzed according to the JIP test. From OJIP, the measured parameters (F_m_, F_20 μs_, F_50 μs_, F_100 μs_, F_300 μs_, F_J_, F_I _etc.) led to the calculation and derivation of a range of new parameters according to previous authors [30,47-51] (see Table 1).

**Table 1:** Summary of parameters, formulae and their description using data extracted from chlorophyll a fluorescence (OJIP) transient.

Fluorescence parameters	Description
Extracted parameters	
F_t_	Fluorescence intensity at time t after onset of actinic illumination
F_50 μs_or F_20 μs_	Minimum reliable recorded fluorescence at 50 μs with the PEA- or 20 μs with Handy-PEA-fluorimeter
F_100 μs _and F_300 μs_	Fluorescence intensity at 100 and 300 μs, respectively
F_J _and F_I_	Fluorescence intensity at the J-step (2 ms) and the I-step (30 ms), respectively
F_P _(= F_m_)	Maximum recorded (= maximum possible) fluorescence at P-step
Area	Total complementary area between fluorescence induction curve and F = F_m_
Derived parameters	
Selected OJIP parameters	
F_o _≅ F_50 μs_or F_o _≅ F_20 μs_	Minimum fluorescence, when all PSII RCs are open
F_m _= F_P_	Maximum fluorescence, when all PSII RCs are closed
V_J _= (F_2 ms _- F_o_)/(F_m _- F_o_)	Relative variable fluorescence at the J-step (2 ms)
V_I _= (F_30 ms _- F_o_)/(F_m _- F_o_)	Relative variable fluorescence at the I-step (30 ms)
M_o _= 4 (F_300 μs _- F_o_)/(F_m _- F_o_)	Approximated initial slope (in ms^-1^) of the fluorescence transient V = f(t)
V_K _= (F_300 μs _- F_o_)/(F_m _- F_o_)	Relative variable fluorescence at 300 μs
S_m _= EC_o_/RC = Area/(F_m _- F_o_)	Normalized total complementary area above the OJIP (reflecting multiple-turnover Q_A _reduction events) or total electron carriers per RC
Fraction of OEC = [1 - (V_K_/V_J_)]_treated sample_/[1 - (V_K_/V_J_)]_control_	The fraction of oxygen evolving centers (OEC) in comparison with control
Yields or flux ratios	
φ_Po _= TR_o_/ABS = 1 - F_o_/F_m _= F_v_/F_m_	Maximum quantum yield of primary photochemistry at t = 0
ψ_Eo _= ET_o_/TR_o _= 1 - V_J_	Probability (at time 0) that a trapped exciton moves an electron into the electron transport chain beyond Q_A_^-^
φ_Do _= DI_o_/ABS = 1 - φ_Po _= F_o_/F_m_	Quantum yield at t = 0 for energy dissipation
δ_Ro _= RE_o_/ET_o _= (1 - V_I_)/(1 - V_J_)	Efficiency with which an electron can move from the reduced intersystem electron acceptors to the PSI end electron acceptors
φ_Ro _= RE_o_/ABS = φ_Po _× ψ_Eo _× δ_Ro_	Quantum yield for the reduction of end acceptors of PSI per photon absorbed
Specific fluxes or activities expressed per reaction center (RC)	
ABS/RC = M_o _× (1/V_J_) × (1/φ_Po_)	Absorption flux per RC
TR_o_/RC = M_o_/V_J_	Trapped energy flux per RC at t = 0
ET_o_/RC = (M_o_/V_J_) × ψ_Eo _= (M_o_/V_J_) × (1 - V_J_)	Electron transport flux per RC at t = 0
DI_o_/RC = ABS/RC - TR_o_/RC	Dissipated energy flux per RC at t = 0
RE_o_/RC = (RE_o_/ET_o_) × (ET_o_/RC)	Reduction of end acceptors at PSI electron acceptor side per RC at t = 0
Phenomenological fluxes or activities expressed per excited cross section (CS)	
DI_o_/CS_o _= ABS/CS_o _- TR_o_/CS_o_	Dissipated energy flux per CS at t = 0
Performance index	
PI_tot,abs _= (RC/ABS) × (φ_Po_/(1 - φ_Po_)) × (ψ_Eo_/(1 - ψ_Eo_)) × (δ_Ro_/(1 - δ_Ro_))	Total performance index, measuring the performance up to the PSI end electron acceptors

### Experimental design and statistical analysis

There were 30 pot seedlings per treatment in a completely randomized design. Experiments were performed with 4 – 15 replicates (one plant from different pots per replicate). Differences among treatments were separated by the least significant difference (LSD) test at *P *< 0.05 level.

## Abbreviations

Al: aluminum; B: boron; Chl: chlorophyll; CS: excited cross section; ET_o_/TR_o_: probability (at time 0) that a trapped exciton moves an electron into the electron transport chain beyond Q_A_^-^; OJIP: Chl a fluorescence; DW: dry weight; PI_tot,abs_: total performance index; F_o_: minimum fluorescence; RC: reaction center; RE_o_/ABS: quantum yield of electron transport from Q_A_^- ^to the PSI end electron acceptors; Rubisco: ribulose-1,5-bisphosphate carboxylase/oxygenase; RuBP: ribulose-1,5-bisphosphate; TR_o_/ABS or F_v_/F_m_: maximum quantum yield of primary photochemistry at t = 0; V_I_: relative variable fluorescence at the I-step; V_J_: relative variable fluorescence at the J-step.

## Authors' contributions

HXJ performed most of the experiments and wrote the manuscript. NT helped in measuring Rubisco activity and OJIP transients. JGZ helped in designing the study. LSC designed and directed the study and revised the manuscript. All authors have read and approved the final manuscript.
